# Participation in and adherence to physical exercise after completion of primary cancer treatment

**DOI:** 10.1186/s12966-016-0425-3

**Published:** 2016-09-09

**Authors:** Caroline S. Kampshoff, Willem van Mechelen, Goof Schep, Marten R. Nijziel, Lenja Witlox, Lisa Bosman, Mai J. M. Chinapaw, Johannes Brug, Laurien M. Buffart

**Affiliations:** 1 Department of Public & Occupational Health, and the EMGO Institute for Health and Care Research, VU University Medical Center, Amsterdam, The Netherlands; 2 Department of Epidemiology and Biostatistics and the EMGO Institute for Health and Care Research, VU University Medical Center, De Boelelaan 1089a, 1081 HV Amsterdam, The Netherlands; 3 Department of Sports Medicine, Máxima Medical Center, Veldhoven, The Netherlands; 4 Department of Hematology, Radboud University Medical Center, Nijmegen, The Netherlands

**Keywords:** Cancer survivors, Exercise, Participation, Exercise adherence, Session attendance, Compliance, Correlates

## Abstract

**Background:**

﻿The purpose of this study was  to identify demographic, clinical, psychosocial, physical and environmental factors that are associated with participation in and adherence to a combined resistance and endurance exercise program among cancer survivors, shortly after completion of primary cancer treatment. Data from the randomized controlled Resistance and Endurance exercise After ChemoTherapy (REACT) study were used for this study.

**Methods:**

The participants of the REACT study were randomly allocated to either a high intensity (HI) or low-to-moderate intensity (LMI) exercise program. Patients’ participation rate was defined as the cancer survivors’ decision to participate in the REACT study. Exercise adherence reflected participants’ attendance to the scheduled exercise sessions and their compliance to the prescribed exercises. High session attendance rates were defined as attending at least 80 % of the sessions. High compliance rates were defined as performing at least of 90 % of the prescribed exercise across all sessions. Correlates of exercise adherence were studied separately for HI and LMI exercise. Demographic, clinical, and physical factors were assessed using self-reported questionnaires. Relevant clinical information was extracted from medical records. Multivariable logistic regression analyses were applied to identify correlates that were significantly associated with participation, high session attendance, high compliance with resistance and high compliance with endurance exercises.

**Results:**

Participants were more likely to have higher education, be non-smokers, have lower psychological distress, higher outcome expectations, and perceive more exercise barriers than non-participants. In HI exercise, higher self-efficacy was significantly associated with high session attendance and high compliance with endurance exercises, and lower psychological distress was significantly associated with high compliance with resistance exercises. In LMI exercise, being a non-smoker was significantly associated with high compliance with resistance exercises and higher BMI was significantly associated with high compliance with resistance and endurance exercises. Furthermore, breast cancer survivors were less likely to report high compliance with resistance and endurance exercises in LMI exercise compared to survivors of other types of cancer. The discriminative ability of the multivariable models ranged from 0.62 to 0.75.

**Conclusion:**

Several demographic, clinical and psychosocial factors were associated with participation in and adherence to exercise among cancer survivors. Psychosocial factors were more strongly associated with adherence in HI than LMI exercise.

**Trial registration:**

This study was registered at the Netherlands Trial Register [NTR2153] on the 5^th^ of January 2010.

## Background

Supervised exercise programs following cancer diagnosis show significant and clinically relevant beneficial effects on cardiorespiratory fitness [1], general and physical fatigue [2] and quality of life (QoL) [3]. More specifically, exercise can improve cancer survivors’ cardiorespiratory fitness, thereby reducing fatigue and improve global QoL and physical function [4]. In addition, observational studies have reported positive associations of physical activity [5] and fitness [6] with cancer-free and overall survival, yet, randomized controlled trials (RCTs) need to establish causality.

The success of RCTs evaluating exercise programs depends largely on patients’ participation and exercise adherence rates. Patients’ participation rate reflects the decision by cancer survivors whether or not to participate in a randomized controlled trial evaluating exercise interventions. Exercise adherence reflects participants’ session attendance rates and their compliance to the prescribed exercises. A better understanding of the modifiable and unmodifiable correlates that are associated with participation in and adherence to exercise interventions may inform future interventions and facilitate successful implementation of exercise programs among cancer survivors. Modifiable correlates (e.g., psychosocial) provide insights into intervention target components via which improvements in participation or adherence might be achieved. Unmodifiable correlates, such as demographics (e.g., age) or clinical variables (e.g., treatment type) indicate which subgroups of patients are most at risk for non-participation or low exercise adherence rates and can thus help to identify relevant target populations for intervention.

Correlates of participation in exercise trials during primary cancer treatment have been investigated by two previous trials [7, 8] and both studies reported that participants who perceived higher levels of fatigue were less likely to participate in the exercise trials. Furthermore, minimizing practical barriers to participation such as travel distances to practices and flexible training schedules were suggested as promising strategies to enhance participation in forthcoming studies [7, 8]. However, correlates of participation in exercise trials after completion of primary cancer treatment have not been studied yet, and may differ from those during primary cancer treatment.

In a recent systematic review, we identified correlates of exercise adherence among cancer survivors [9], and found exercise history to be significantly associated with exercise adherence. Other important demographic, clinical, psychosocial and environmental correlates of exercise adherence could not be distinguished due to the limited number of studies, or the inconsistency of findings across the reviewed manuscripts. Moreover, the definition of exercise adherence varied across the reviewed studies. Some studies exclusively focused on session attendance rate, while other studies also incorporated a measure on compliance. Therefore, more research is warranted.

The current study aimed to identify demographic, clinical, psychosocial, physical and environmental factors that are associated with participation in an exercise program and exercise adherence among cancer survivors, shortly after completion of primary cancer treatment. We used data of the Resistance and Endurance exercise After ChemoTherapy (REACT) study [10], a RCT that evaluated the effectiveness of a high intensity (HI) and low-to-moderate intensity (LMI) exercise compared to a waiting list control (WLC) group shortly after completion of primary cancer treatment on physical fitness, fatigue and health-related quality of life (HRQoL). We found that HI and LMI exercise significantly improved cardiovascular fitness, reduced fatigue and improved quality of life [11].

## Methods

Detailed procedures of the REACT study have been reported elsewhere [11]. Briefly, the REACT study was a multicenter RCT in which 277 cancer survivors were randomized into three study arms: HI exercise, LMI exercise, and a WLC group. Between 2011 and 2013, patients were recruited from 9 hospitals in the Netherlands. Patients aged ≥ 18 years with histologically confirmed breast, colon, ovarian, cervix or testis cancer, or lymphomas with no indication of recurrent or progressive disease, who had completed ((neo-) adjuvant) chemotherapy were eligible. Patients were excluded if they were unable to perform basic physical activity, had cognitive disorders, severe emotional instability, comorbidities that might hamper capacity of carrying out HI exercise, or were unable to understand and read the Dutch language. This study was approved by the Medical Ethics Committee of the VU University Medical Centre [2011/240] and the local ethical boards of all participating hospitals.

### (Non-)participation

Patients who were willing to participate were invited for baseline measurements, 4–6 weeks after completion of primary cancer treatment. After baseline measurements, participants were stratified by cancer type and hospital, and randomly assigned to one of the three study arms. HI and LMI groups started with their 12-weeks exercise program directly after randomization (i.e., direct start). Participants from the WLC group were also randomly allocated to HI or LMI exercise. However, they started exercising 12 weeks later. Patients who chose not to participate (i.e., non-participants) were invited to complete a one-time survey that was similar in content and timing to the baseline questionnaire of the REACT participants. Written informed consent was obtained from both non-participants and participants, including permission to extract relevant information from their medical records. Finally, from patients who chose to refrain from any participation, only age, gender and cancer type were documented.

#### Exercise interventions

Full details of the 12-week HI and LMI programs have been described previously [10]. In short, both interventions included two one-hour supervised resistance and endurance exercise sessions per week and were identical with respect to exercise frequency, type, and duration, and differed only in exercise intensity (Table 1). Both exercise programs included six resistance exercises targeting large muscle groups with a frequency of two sets of ten repetitions. Workload per exercise was defined by an indirect one repetition maximum (1-RM) measurement. Furthermore, both programs included two types of endurance interval exercises, aiming to maximize improvements in cardiorespiratory fitness. In the first four weeks patients cycled 2x8 min with alternating workloads. Workloads were defined by the maximum short exercise capacity (MSEC) estimated by the steep ramp test. From the fifth week onwards, one additional endurance interval session was added, substituting eight minutes of cycling. This interval session consisted of three times five minutes cycling at a constant workload. Here, the workload was defined by the heart rate reserve (HRR), using the Karvonen formula. Twenty-one local physiotherapists supervised all training sessions. In the Netherlands, people are generally used to short travel distances to their health care providers and therefore patients trained at local physiotherapists practices close to the patients’ homes. The availability of flexible training hours and the possibility to join a rehabilitation group differed per practice. Furthermore, the start of the exercise programs was linked to the time point of completion of the primary cancer treatment of the individual cancer survivor. Consequently, the training hours and the availability of group sessions varied.

**Table 1:** Exercise intensities of the HI and LMI resistance and endurance exercise programs

	Resistance exercises (1-RM)^a^ (10 repetitions in 2 series of 6 exercises targeting the large muscle groups)	Endurance interval exercises Part A (MSEC)^a^ (8 min alternating workload)	Endurance interval exercises Part B (HRR)^a^ (3x5 min constant workload)	Counseling
High intensity (HI) exercise^b^	70-85 %	30/65 %	≥80 %	Participants were encouraged to start or maintain a physically active lifestyle in addition to the supervised exercise sessions.
Low-to-moderate intensity (LMI) exercise^b^	40-55 %	30/45 %	40-50 %	

*Abbreviations*: *1-RM* one repetition maximum, *MSEC* maximum short exercise capacity, *HRR* heart rate reserve, *HI* high intensity exercise, *LMI* low-to-moderate intensity exercise

^a^Every four weeks (week 1, 5 and 9), the physiotherapist evaluated training progress, and adjusted the workload accordingly. ^b^Exercises were accompanied with BORG scores and heart rate monitors to guide the physiotherapists. In the occasion that the training intensity seemed too high or too low, the 1-RM, MSEC or HRR were reassessed

#### Adherence

Adherence was defined as attendance to the prescribed number of sessions and compliance with the prescribed intensity, frequency and duration of the prescribed resistance and endurance exercises [12, 13]. Both session attendance rates and compliance rates were retrieved from exercise logs completed by the physiotherapists. *Session attendance* was defined as the number of supervised exercise sessions attended, divided by the number of supervised exercise sessions offered. *Compliance with resistance exercises* was defined in terms of intensity and volume (Table 2), in which compliance with the intensity of the resistance exercises was calculated by the performed training load, divided by the prescribed training load and compliance with the volume of the resistance exercises was calculated by the performed number of repetitions, divided by the prescribed number of repetitions. The average value of compliance with intensity and volume provided the overall measure for compliance with the resistance exercises. *Compliance with endurance exercises* was defined as exercise duration (in minutes), divided by the prescribed exercise duration (Table 2). The average of this parameter provided the overall measure for compliance with the endurance exercises. Next, the normality assumption was tested for *session attendance*, *compliance with resistance training*, and *compliance with endurance training.* Since they were skewed, and to facilitate clinically meaningful interpretation, we dichotomized adherence outcome variables based on clinically-relevant cut-off points. In line with previous studies, *high session attendance* was defined as attending at least 80 % of the sessions [14]. We defined *high compliance* rates as performing at least 90 % of the resistance and endurance exercises according to the prescribed dosage. This cut-off point of 90 % allowed some deviation due to the rounded weights and settings of the local training equipment, while maintaining a sufficient distinction between HI and LMI exercise.

**Table 2:** Outcome measures of compliance to the prescribed exercises

Compliance		
Resistance exercises		Endurance interval exercises
Intensity	Volume	Duration
$\frac{\mathrm{Used}\kern0.5em {\mathrm{load}}^{\mathrm{a}}}{\mathrm{Prescribed}\kern0.5em \mathrm{load}}\kern0.5em \times 100\%$	$\frac{\mathrm{Performed}\kern0.5em \mathrm{repetitions}}{\mathrm{Prescribed}\kern0.5em \mathrm{repetitions}}\times 100\%$	$\frac{\mathrm{Performed}\kern0.5em {\mathrm{duration}}^{\mathrm{b}}}{\mathrm{Prescribed}\kern0.5em \mathrm{duration}}\times 100\%$

^a^Load in kilograms, ^b^time in minutes

### Assessment of correlates

Demographic data were collected using a self-report questionnaire and included age at baseline (in years), gender (0 = male; 1 = female), marital status (0 = no partner; 1 = married or de facto), education (0 = low/intermediate; 1 = high), employment status (0 = no paid employment; 1 = paid employment), smoking status (0 = non-smoker; 1 = smoker) and sport history (0 = no; 1 = yes). Furthermore, participants’ travel distance to the exercise program (in kilometres) was calculated based on zip codes of the patient’s home and location of training facility.

Clinical information was retrieved from medical records and included cancer type (0 = breast cancer; 1 = other (i.e., colon, ovarian, cervix or testis cancer, or lymphomas)), stage of disease (0 = stage I-II, 1 = stage III-IV), previous treatment with surgery (0 = no; 1 = yes), radiation therapy (0 = no; 1 = yes), immunotherapy (0 = no; 1 = yes), hormone therapy (0 = no; 1 = yes), and two or more of the following comorbidities (0 = no; 1 = yes) including heart disease, lung disease, diseases of the digestive system, diseases of the nervous system, endocrine disease, mental disorder, rheumatism or arthritis, or chronic pain [15]. In addition, body weight of the REACT participants was measured to the nearest 0.1 kg on a digital scale, with light clothes on and no shoes. Body height was measured to the nearest 0.1 cm without shoes. Body mass index (BMI) was calculated from the measured body weight and height accordingly.

Patient-reported outcomes have been reported elsewhere [16] and included general fatigue (subscale of the Multidimensional Fatigue Inventory (MFI) [17]), global quality of life (subscale of the European Organisation Research and Treatment of Cancer - Quality of Life Questionnaire C30 (EORTC QLQ C-30) [18]), psychological distress (Hospital Anxiety and Depression Scale (HADS) [19]) and self-reported physical activity using the Physical Activity Scale for the Elderly questionnaire (PASE) [20].

Patient-reported behavioral and attitudinal factors towards exercise included a series of questions that were based on health behavior theories, in particular the Theory of Planned Behavior [21]. Current attitude towards exercise participation was measured by one item ‘In my opinion regular exercise is..’ rated on 5-point Likert scale (1 = very bad to 5 = very good) [21]. Barriers to exercise behavior (Cronbach’s α = 0.85) were measured using 18 items (e.g., my disease, insufficient motivation, lack of energy), rated on 5-point Likert scale (1 = never to 5 = very often) [21, 22]. Outcome expectations regarding exercise participation (Cronbach’s α = 0.91) included 12 items (e.g., increase my health, feel better about myself, and be more physically fit), rated on a 5-point Likert scale (1 = strongly disagree to 5 = strongly agree) [21, 22]. Exercise self-efficacy (Cronbach’s α = 0.83) was assessed with the following question ‘How confident are you that you will be physically active in the following situations’, including feeling tired, bad mood, do not have the time, on vacation, and, want to be active outside, but bad weather, rated on a 10-point Likert scale (1 = absolutely no confidence, 10 = completely confident) [23]. Social support for exercise (Cronbach’s α = 0.92) was assessed using the statement: ‘The following people are supportive of my regular PA’, followed by: family, friends, and other cancer patients, rated on a 5-point Likert scale (1 = strongly disagree to 5 = strongly agree) [24]. Mean scores were calculated for potential correlates that comprised more than one item. After that, all factors were linearly transformed to a 0-100 scale. Exercise stage was measured using the response options derived from the Transtheoretical Model [25]: (1) no intention to exercise; (2) intention to exercise; (3) irregular exercise; (4) started exercising 30 min a day in last 6 months; or (5) exercising 30 min a day for longer than 6 months, that were dichotomized into non-exerciser (response options 1–3) and exerciser (response options 4–5).

#### Statistical analyses

The statistical analyses were performed using IBM SPSS Statistics (SPSS Inc., Evanston, IL, version 22.0). Descriptive statistics (mean and standard deviations (SD)) were calculated for all outcome variables. Data on BMI and travel distance to the exercise program for the non-participants were not available due to trial logistics. Correlations between all potential correlates were checked for multicollinearity (r ≥ 0.60). Multicollinearity was present between cancer type and gender. Because cancer type was most strongly associated with exercise adherence, this variable was included in the model instead of gender. Differences in session attendance rates and compliance rates between HI and LMI exercise were tested using chi-square tests. To examine whether correlates of exercise adherence differed between HI and LMI exercise groups, we added an interaction term of the correlate with the interventions into a regression model, separately for each correlate. As significant interaction terms were found for education, cancer type, self-efficacy, and psychological distress, we performed stratified analyses for HI and LMI. All analyses on exercise adherence were performed according to an intention-to-treat principle.

Univariable and multivariable logistic regression analyses were conducted to identify factors that were significantly correlated with participation and exercise adherence. Separate multivariable logistic regression analyses with a forward selection procedure were carried out for each outcome variable: participation, high session attendance and high compliance with the resistance and endurance exercises. By default, timing of intervention (i.e., direct start or WLC group) was retained as covariate in the univariable and multivariable models of exercise adherence. First the independent variables with a *p*-value ≤0.25 in the univariable analyses were selected for further analyses. After that, a multivariable stepwise forward selection procedure was undertaken by identifying the correlates that was most strongly associated with the dependent variable. Subsequently, the next strongest related correlate was then selected after controlling for the first correlate. Only variables with a *p*-value of ≤0.05 were retained in the final multivariable model. The regression coefficients (β) and odds ratio (OR) with 95 % CI were reported accordingly. In addition, the model fit was evaluated by the area under the receiver operating characteristic curve (AUC) with 95 % CI.

## Results

In total, 277 out of 757 eligible patients (37 %) participated. Furthermore, 179 patients (24 %) did not participate in the trial, but completed the one-time survey (i.e., non-participants). Self-reported reasons for non-participation were having too many things on one’s mind (*n* = 72), already exercising (*n* = 30), not wanting to be randomized (*n* = 20), and not interested to participate in a clinical trial (*n* = 13). For 44 non-participants the reason of non-participation was unknown. Baseline demographic, clinical, psychosocial and physical characteristics of the participants and non-participants are presented in Table 3. 90 % of the participants and non-participants underwent surgery to treat cancer, revealing very little variability within our population. Therefore, we omitted surgery as a potential correlate for participation and exercise adherence from the multivariable regression analyses.

**Table 3:** Baseline characteristics of (non-) participants

	Non-participants (*n* = 179)	Participants (*n* = 277)	
		HI (*n* = 139)	LMI (*n* = 138)
Demographic			
Age, mean (SD) years	55 (10.6)	54 (10.7)	53 (11.4)
Gender, *n* (%) male	26 (15)	29 (21)	26 (19)
Marital status, *n* (%) having a partner	150 (84)	112 (81)	120 (87)
Education, *n* (%) high	43 (25)	52 (38)	53 (39)
Employment status at baseline, *n* (%) yes	91 (51)	85 (61)	82 (59)
Smoking status at baseline, *n* (%) yes	27 (15)	9 (7)	8 (6)
Sport history, *n* (%) yes	100 (57)	72 (52)	83 (61)
Clinical			
Cancer type, *n* (%)			
Breast	120 (67)	92 (66)	89 (65)
Colon	31 (17)	25 (18)	24 (17)
Ovarian	6 (3)	8 (6)	4 (3)
Lymphoma	16 (9)	10 (7)	16 (12)
Cervix	4 (2)	0	4 (3)
Testis	2 (1)	4 (3)	1 (1)
Stage of disease, *n* (%)			
Local	123 (69)	103 (74)	84 (61)
Advanced	55 (31)	36 (26)	54 (39)
Type of treatment, *n* (%)			
Surgery	161 (90)	127 (91)	123 (89)
Radiation therapy	87 (49)	74 (53)	61 (44)
Immunotherapy	33 (18)	23 (17)	36 (26)
Hormone therapy	81 (45)	67 (48)	61 (44)
Comorbidities ≥2, *n* (%) yes	26 (15)	16 (12)	14 (10)
BMI in kg/m^2^, mean (SD)	n.a.	27.2 (4.5)	26.6 (4.2)
Psychosocial			
General fatigue (MFI), mean (SD)^a^	12.3 (4.4)	12.8 (3.8)	12.9 (4.2)
Global HRQoL (QLQ-C30), mean (SD)^b^	70.9 (18.2)	71.3 (16.2)	73.1 (16.1)
Psychological distress (HADS), mean (SD)^c^	8.2 (6.4)	7.4 (5.7)	7.6 (5.3)
Attitude, mean (SD)	83.8 (21.2)	85.2 (22.3)	87.3 (18.8)
Perceived barriers, mean (SD)	23.1 (12.4)	28.6 (12.3)	25.8 (12.0)
Outcome expectations, mean (SD)	71.8 (17.8)	75.0 (15.7)	76.0 (13.7)
Self-efficacy, mean (SD)	64.8 (17.9)	59.9 (16.6)	61.1 (16.4)
Social support, mean (SD)	79.3 (21.1)	81.9 (20.6)	81.0 (20.1)
Physical			
Exercise stage, *n* (%)^d^	85 (50)	66 (49)	67 (49)
Self-reported PA	115.6 (91.3)	96.7 (69.0)	106.2 (83.1)
Environmental			
Travel distance to the exercise program (in kilometres), mean (SD)	n.a.	7.3 (6.1)	6.5 (4.8)
Adherence			
High session attendance, *n* (%)	-	106 (76)	93 (67)
High compliance with resistance exercises, *n* (%)	-	93 (69)	87 (67)
High compliance with endurance exercises, *n* (%)	-	64 (47)	55 (42)

*Abbreviations*: *SD* standard deviation, *n* number, *HI* high intensity exercise, *LMI* low-to-moderate intensity exercise, *BMI* body mass index, *n.a.* not applicable, *PA* physical activity

^a^Range 4-20, higher score means a higher level of self-reported general fatigue; ^b^Range 0-100, higher score means a higher level of self-reported global HRQoL; ^c^Range 0-36, higher means a higher level of anxiety and/or depression; ^d^currently exercising

*High session attendance* was found in 76 % and 67 % of the participants in HI and LMI groups respectively (*p* = 0.10). *High compliance with resistance exercises* was found in 69 % of the participants in HI and in 67 % of participants in LMI (*p* = 0.80). *High compliance with endurance exercises* was found in 47 % of the participants in HI and in 42 % of the participants in LMI (*p* = 0.40).

### Correlates of participation

The results of the univariable and multivariable logistic regression analyses are presented in Table 4. The multivariable regression model showed that participants were more likely to have higher education, (OR = 1.79, 95 % CI: 1.14; 2.82), non-smoking habits (OR = 0.46, 95 % CI: 0.23; 0.92), lower psychological distress (OR = 0.94, 95 % CI: 0.91; 0.98), higher outcome expectations (OR = 1.02, 95 % CI: 1.01; 1.04), and perceive more exercise barriers (OR = 1.05, 95 % CI: 1.03; 1.07) than non-participants. The AUC for this model was 0.69 (95 % CI: 0.64; 0.74). No significant associations were found between participation and treatment-related or physical characteristics.

**Table 4:** Odds ratios and their 95 % confidence intervals as results from univariable and multivariable logistic regression analyses with participation as dependent variable and demographic, clinical, psychosocial and physical variables as independent variables

	Univariable, OR (95 % CI)	Multivariable, OR (95 % CI)
Demographic		
Age	0.99 (0.97;1.00)	
Gender	0.69 (0.41;1.14)	
Marital status	1.00 (0.60;1.66)	
Education	**1.89 (1.24;2.89)**	**1.79 (1.14;2.82)**
Employment status	**1.47 (1.01;2.15)**	
Smoking status	**0.37 (0.19;0.70)**	**0.46 (0.23;0.92)**
Sport history	0.99 (0.67;1.44)	
Clinical		
Cancer type	1.08 (0.73;1.61)	
Stage of disease	1.08 (0.72;1.61)	
Type of treatment		
Radiation therapy	1.01 (0.69;1.46)	
Immunotherapy	1.20 (0.75;1.93)	
Hormone therapy	1.04 (0.71;1.52)	
Comorbidities (≥2)	0.72 (0.41;1.25)	
Psychosocial		
General fatigue	1.03 (0.99;1.08)	
Global HRQoL	1.01 (0.99;1.02)	
Psychological distress	0.98 (0.95;1.01)	**0.94 (0.91;0.98)**
Attitude	1.01 (1.00;1.02)	
Perceived barriers	**1.03 (1.01;1.05)**	**1.05 (1.03;1.07)**
Outcome expectations	**1.02 (1.00;1.03)**	**1.02 (1.01;1.04)**
Self-efficacy	**0.99 (0.97;1.00)**	
Social support	1.01 (1.00;1.01)	
Physical		
Exercise stage	0.97 (0.66;1.42)	
Self-reported PA	1.00 (1.00;1.00)	

*Abbreviations*: *p* ≤ 0.05 in bold, *OR* odds ratio, *CI* confidence interval, *HRQoL* Health-related quality of life, *PA* physical activity

### Correlates of adherence in HI

In HI, higher self-efficacy was significantly associated with *high session attendance* (OR = 1.06, 95 % CI: 1.03; 1.09; AUC = 0.75, 95 % CI: 0.66; 0.84) and *high compliance with endurance exercises* (OR = 1.05, 95 % CI: 1.02; 1.07; AUC = 0.68, 95 % CI: 0.59; 0.77) (Table 5). Furthermore, less psychological distress (OR = 0.87, 95 % CI: 0.81; 0.94) was significantly correlated with *high compliance with resistance exercises* in HI (AUC = 0.69, 95 % CI: 0.60; 0.79). Demographic, treatment-related, physical and environmental characteristics were not significantly associated with exercise adherence in HI.

**Table 5:** Odds ratios and their 95 % confidence intervals as results from univariable and multivariable logistic regression analyses with session attendance and compliance with the resistance and endurance exercises as dependent variables and demographic, clinical, psychosocial and physical variables as independent variables

	HI						LMI					
	Session attendance		Compliance with the resistance exercises		Compliance with the endurance exercises		Session attendance		Compliance with the resistance exercises		Compliance with the endurance exercises	
	Univariable, OR (95 % CI)	Multivariable, OR (95 % CI)	Univariable, OR (95 % CI)	Multivariable, OR (95 % CI)	Univariable, OR (95 % CI)	Multivariable, OR (95 % CI)	Univariable, OR (95 % CI)	Multivariable, OR (95 % CI)	Univariable, OR (95 % CI)	Multivariable, OR (95 % CI)	Univariable, OR (95 % CI)	Multivariable, OR (95 % CI)
Demographic												
Age	1.01 (0.98;1.05)		1.00 (0.97;1.03)		1.01 (0.97;1.04)		1.00 (0.97;1.04)		1.03 (0.99;1.06)		1.01 (0.98;1.05)	
Gender	0.62 (0.22;1.78)		0.51 (0.19;1.37)		0.97 (0.42;2.20)		0.71 (0.27;1.83)		0.45 (0.16;1.30)		0.41 (0.17;1.01)	
Marital status	1.15 (0.44;3.04)		1.22 (0.49;3.02)		1.29 (0.54;3.07)		0.70 (0.23;2.14)		0.64 (0.19;2.16)		0.40 (0.14;1.20)	
Education	1.29 (0.56;2.94)		1.84 (0.84;4.05)		1.67 (0.83;3.38)		0.66 (0.32;1.37)		0.49 (0.23;1.05)		**0.45 (0.21;0.94)**	
Employment status	1.66 (0.75;3.66)		0.96 (0.45;2.03)		1.38 (0.69;2.78)		0.84 (0.41;1.75)		**0.43 (0.19;0.94)**		0.67 (0.33;1.35)	
Smoking status	0.62 (0.14;2.64)		1.66 (0.33;8.42)		0.90 (0.23;3.52)		0.27 (0.06;1.19)		**0.18 (0.03;0.97)**	**0.16 (0.03;0.91)**	0.22 (0.03;1.87)	
Sport history	1.61 (0.73;3.55)		0.95 (0.46;1.98)		1.22 (0.62;2.41)		1.40 (0.67;2.91)		1.54 (0.72;3.28)		0.92 (0.45;1.89)	
Clinical												
Cancer type	1.21 (0.52;2.81)		1.90 (0.83;4.35)		0.94 (0.46;1.93)		2.16 (0.97;4.80)		**2.71 (1.16;6.36)**	**3.25 (1.31;8.02)**	**2.66 (1.27;5.56)**	**2.94 (1.38;6.27)**
Stage of disease	1.39 (0.54;3.56)		1.73 (0.71;4.22)		0.77 (0.36;1.69)		0.82 (0.40;1.69)		0.85 (0.40;1.82)		1.30 (0.63;2.66)	
Type of treatment												
Radiation therapy	2.04 (0.92;4.54)		0.79 (0.38;1.65)		1.39 (0.71;2.76)		0.99 (0.49;2.04)		0.90 (0.43;1.88)		1.17 (0.58;2.37)	
Immunotherapy	0.67 (0.25;1.82)		1.05 (0.40;2.79)		1.96 (0.78;4.91)		0.58 (0.26;1.28)		0.85 (0.37;1.93)		1.11 (0.50;2.44)	
Hormone therapy	1.17 (0.53;2.58)		0.79 (0.38;1.64)		1.31 (0.67;2.59)		1.32 (0.64;2.72)		0.58 (0.28;1.23)		0.69 (0.34;1.41)	
Comorbidities	0.68 (0.22;2.12)		0.66 (0.22;1.99)		0.73 (0.24;2.17)		0.63 (0.20;1.96)		0.75 (0.23;2.48)		1.62 (0.51;5.18)	
BMI	0.93 (0.86;1.02)		0.99 (0.91;1.08)		0.94 (0.87;1.02)		1.05 (0.96;1.15)		1.10 (0.96;1.22)	**1.11 (1.00;1.23)**	1.09 (1.00;1.19)	**1.11 (1.01;1.21)**
Psychosocial												
General fatigue	0.92 (0.83;1.02)		0.92 (0.83;1.01)		0.97 (0.89;1.06)		1.00 (0.92;1.09)		1.03 (0.94;1.13)		1.05 (0.96;1.14)	
Global HRQoL	1.01 (0.99;1.03)		1.02 (1.00;1.04)		1.01 (0.98;1.03)		1.00 (0.98;1.02)		1.00 (0.98;1.02)		1.01 (0.99;1.03)	
Psychological distress	**0.93 (0.87;0.99)**		**0.87 (0.81;0.94)**	**0.87 (0.81;0.94)**	**0.91 (0.85;0.98)**		1.00 (0.94;1.07)		1.01 (0.94;1.08)		1.00 (0.93;1.07)	
Attitude	**1.03 (1.01;1.04)**		**1.02 (1.01;1.04)**		**1.02 (1.00;1.04)**		1.01 (0.99;1.03)		1.00 (0.98;1.02)		1.01 (0.99;1.03)	
Perceived barriers	**0.96 (0.92;0.99)**		**0.97 (0.94;1.00)**		0.98 (0.95;1.01)		0.99 (0.96;1.02)		0.98 (0.95;1.01)		0.98 (0.95;1.01)	
Outcome expectations	1.01 (0.99;1.04)		1.00 (0.98;1.03)		1.01 (0.99;1.03)		1.00 (0.98;1.03)		1.02 (0.99;1.05)		1.00 (0.98;1.03)	
Self-efficacy	**1.06 (1.03;1.09)**	**1.06 (1.03;1.09)**	1.02 (1.00;1.05)		**1.05 (1.02;1.07)**	**1.05 (1.02;1.07)**	1.00 (0.98;1.02)		1.00 (0.98;1.03)		1.01 (0.99;1.03)	
Social support	1.00 (0.98;1.02)		1.00 (0.99;1.02)		1.00 (0.98;1.02)		1.00 (0.98;1.02)		1.00 (0.98;1.02)		1.01 (0.99;1.02)	
Physical												
Exercise stage	**2.96 (1.26;6.93)**		1.08 (0.52;2.26)		1.66 (0.83;3.33)		0.86 (0.42;1.77)		1.23 (0.58;2.60)		0.65 (0.32;1.32)	
Self-reported PA	1.00 (0.99;1.01)		1.00 (1.00;1.01)		1.00 (1.00;1.01)		1.00 (0.99;1.00)		1.00 (0.99;1.00)		1.00 (1.00;1.00)	
Environmental												
Travel distance to the exercise program	1.03 (0.96;1.11)		0.95 (0.89;1.03)		0.99 (0.92;1.06)		1.00 (0.94;1.07)		1.06 (0.99;1.15)		1.02 (0.96;1.08)	

*Abbreviations*: *p* ≤ 0.05 in bold, *OR* odds ratio, *CI* confidence interval, *HRQoL* Health-related quality of life, *BMI* body mass index, *PA* physical activity

### Correlates of adherence in LMI

In LMI, being a non-smoker (OR = 0.16, 95 % CI: 0.03; 0.91) was significantly associated with *high compliance with resistance exercises* (Table 5), and higher BMI was significantly associated with *high compliance with resistance exercises* (OR = 1.11, 95 % CI: 1.00; 1.23) and *endurance exercises* (OR = 1.11, 95 % CI: 1.01; 1.21). Furthermore, breast cancer survivors were less likely to report *high compliance with resistance exercises* (OR = 3.25, 95 % CI: 1.31; 8.02) and *high compliance with endurance exercises* (OR = 2.94, 95 % CI: 1.38; 6.27) in LMI than survivors of other types of cancer. The AUC for the models of *high compliance with resistance and endurance exercises* were 0.69 (95 % CI: 0.60; 0.79) and 0.67 (95 % CI: 0.58; 0.77), respectively. Treatment-related, psychosocial, physical and environmental characteristics were not significantly associated with exercise adherence in LMI.

## Discussion

The current study identified important demographic, clinical, psychosocial, physical and environmental factors that may influence participation and exercise adherence, aiming to facilitate successful exercise participation among cancer survivors. We found that some demographic and psychosocial factors were significantly associated with exercise participation and adherence. Additionally, we found that psychosocial factors such as psychological distress and self-efficacy were more strongly associated with adherence to HI than LMI.

### (Non-) participation

The current participation rates of 37 % are in line with previous exercise trials among cancer survivors, reporting that 35–50 % of the eligible patients participated [26, 27]. Our finding that patients with a high level of education were more likely to participate supports previous findings in cancer survivors during active cancer treatment [8]. Accordingly, it has repeatedly been found in non-clinical populations that people who attained higher education are more likely to participate in health behavior change interventions [28]. Furthermore, the current study identified non-smoking as a significant correlate of participation. This was in contrast with a study in the Netherlands, who found no significant association between smoking and participation in an exercise trial during cancer treatment. Possibly, cancer survivors who chose to participate in an exercise trial after completion of primary cancer treatment might have experienced a ‘teachable moment’, or a need to change, during cancer treatment, including quitting smoking and participating in an exercise program [29]. Finally, we found that lower psychological distress, higher outcome expectations and experiencing more exercise barriers were significantly associated with participation. Our findings that patients with higher psychological distress and lower outcome expectations were more likely to decline participation, is in line with the previous study evaluating exercise programs in breast cancer survivors during chemotherapy [8]. Aiming to successfully target those subgroups of patients, previous studies suggested that clinical practice may benefit from behavior change strategies such as motivational interviewing [30]. Yet, further evidence is needed to determine which approaches are most efficacious among cancer survivors. Our finding that REACT participants reported more exercise barriers than non-participants seems paradoxical. However, it is possible that non-participants were not interested in exercise shortly after completion of primary cancer treatment and consequently perceived fewer barriers to obtain and maintain exercise, or that participants were more open to support from healthcare professionals in overcoming their exercise barriers, compared to the non-participants.

### Adherence

In the HI and LMI groups, 76 % and 67 % of the participants showed high attendance rates, which is within the range reported by other exercise trials following cancer diagnosis [9]. However, comparing studies is limited by the scarcity of studies reporting on session attendance rates to supervised exercise after primary cancer treatment [9]. The compliance rates with the resistance and endurance exercises did not differ significantly between HI and LMI. This suggests that the exercise prescriptions were equally feasible to perform when the participants attended the session. Regarding exercise types, compliance rates with resistance exercises were higher than compliance rates with endurance exercises in both groups. This may suggest that resistance exercises are more feasible for cancer survivors than endurance exercises. However, it may also reflect a lower accuracy of defining the maximum workload by MSEC and HRR for the endurance exercises, compared to the 1-RM measurement for the resistance exercises. Although the steep ramp test is a short maximal exercise capacity test, which has proven to be a reliable and valid method to estimate cardiorespiratory fitness in cancer survivors [31], its anaerobic nature may have overestimated the workload for the endurance exercises. Comparably, the Karvonen formula including HRR is a commonly used method to calculate training workload for endurance exercises [32], however, someone’s resting heart rate is prone to day-to-day fluctuations.

Psychosocial variables were significantly associated with *high session attendance* and *high compliance with resistance and endurance exercises* in HI, but not in LMI. This suggests that an individual’s self-efficacy and distress levels are important characteristics while accomplishing a HI exercise program. Hence, including behavioral motivational strategies aiming to improve these psychosocial variables may support cancer survivors in achieving their exercise goals, especially for participants with less favorable scores in these variables to begin with. Participants with less favorable scores could also be recommended to start with LMI exercise, and -after gaining further confidence in exercising- the exercise intensity could gradually increase over time [33].

In LMI, only being a non-smoker or clinical factors were significantly associated with high compliance with the resistance or endurance exercises. Previous studies in non-clinical populations have suggested that health-related behaviors such as a physically active lifestyle and being a non-smoker tend to cluster [34]. This may explain why non-smokers had higher compliance rates. In contrast, the significant association between higher BMI and high compliance with resistance and endurance exercises seems counterintuitive but may indicate that participants with a lower BMI generally had better physical health and found the training intensity of the LMI exercise program is less challenging. A similar explanation could be suggested for breast cancer survivors; LMI exercise might have been too low for them compared to the other five cancer diagnosis with generally lower 5-year survival rates, contributing to lower compliance rates. Though, previous studies also report that breast cancer survivors are more likely to experience difficulties in accomplishing resistance and endurance exercises due to a limited range of motion in the shoulders after surgery [35], cardiorespiratory problems after radiation therapy [36] or joint stiffness as a result of hormone therapy [37]. Yet, in our data we found no significant associations between type of treatment and exercise adherence. Future insight in the role of cancer treatment in exercise adherence is warranted.

### Strengths and limitations

To the best of our knowledge, the current study is the first study assessing correlates of participation in exercise after completion of primary cancer treatment, facilitated by an extensive non-responder questionnaire completed by 179 non-participants. In addition, we assessed factors associated with participants’ exercise adherence taking into account both session attendance, as well as compliance to the prescribed exercises. We included large sample sizes, a relatively large number of potential demographic, clinical, psychosocial and physical correlates, allowing multivariable regression analyses. Yet, the following limitations should be taken into account. First, 301 of our non-participants (63 %) did not complete the extensive non-responder survey, which limits the generalizability of the current findings. Nevertheless, no significant differences in age, gender and cancer type were found between the participants of the one-time survey and the non-responders. Second, the discriminative ability of the models was moderate, ranging from 0.62 to 0.75 [38]. This indicates that there may be other variables that were not included in our study that are important to explain differences in participation and adherence rates among cancer survivors. For example, previous research showed that low socioeconomic status was negatively associated with adherence rates in cardiopulmonary rehabilitation [39] and may warrant further investigation among cancer survivors. Moreover, in general, studies that have investigated social and environmental correlates of participation and adherence rates among cancer survivors are scarce. Previous studies in the healthy population showed that social and environmental factors including peer support, physician influence, and access to facilities at flexible time points [40] were significantly associated with exercise participation. Therefore, future studies should examine whether social and environmental factors are associated with participation in and exercise adherence to an exercise program among cancer survivors. Finally, theory-based interventions have shown to be more effective in changing behavior than non-theory based interventions [41]. Since the current study showed significant associations of outcome expectations and self-efficacy with participation in or adherence to an exercise program, forthcoming studies might consider a role for behavioral theory such as social cognitive theory or self-determination theory [42], to facilitate a better understanding of exercise behavior in cancer survivors.

## Conclusion

This study showed that cancer survivors who attained a higher level of education, were non-smokers, perceived less psychological distress, had higher outcome expectations and perceived more exercise barriers were more likely to participate in a combined resistance and endurance exercise trial. This is worth acknowledging when promoting exercise participation as part of usual cancer care. Furthermore, the current study found several demographic, clinical and psychosocial factors to be significantly associated with exercise adherence in which, psychosocial factors, such as psychological distress and self-efficacy were more strongly associated with HI than LMI exercise. When offering HI exercise, it may therefore be recommended to screen these variables, and if needed, include additional behavioral motivational strategies or consider starting at a lower training intensity.

## Abbreviations

AUC: Area under the receiver operating characteristic curve
BMI: Body mass index
CI: Confidence interval
EORTC QLQ C-30: European Organisation Research and Treatment of Cancer - Quality of Life Questionnaire C30
HADS: Hospital anxiety and depression scale
HI: High intensity
HRQoL: Health-related quality of life
LMI: Low-to-moderate intensity
MFI: Multidimensional fatigue inventory
OR: Odds ratio
PA: Physical activity
PASE: Physical Activity Scale for the Elderly Questionnaire
QoL: Quality of life
RCT: Randomized controlled trial
REACT: Resistance and endurance exercise after chemotherapy
WLC: Waiting list control
